# Intrinsic differentiation potential of adolescent human tendon tissue: an in-vitro cell differentiation study

**DOI:** 10.1186/1471-2474-8-16

**Published:** 2007-02-23

**Authors:** Marieke de Mos, Wendy JLM Koevoet, Holger Jahr, Monique MA Verstegen, Marinus P Heijboer, Nicole Kops, Johannes PTM van Leeuwen, Harrie Weinans, Jan AN Verhaar, Gerjo JVM van Osch

**Affiliations:** 1 Department of Orthopaedics, Erasmus MC, University Medical Centre Rotterdam, POBox 2040, 3000 CA, the Netherlands; 2 Department of Otorhinolaryngology, Erasmus MC, University Medical Centre Rotterdam, POBox 2040, 3000 CA, the Netherlands; 3 Department of Haematology, Erasmus MC, University Medical Centre Rotterdam, POBox 2040, 3000 CA, the Netherlands; 4 Department of Internal Medicine, Erasmus MC, University Medical Centre Rotterdam, POBox 2040, 3000 CA, the Netherlands

## Abstract

**Background:**

Tendinosis lesions show an increase of glycosaminoglycan amount, calcifications, and lipid accumulation. Therefore, altered cellular differentiation might play a role in the etiology of tendinosis. This study investigates whether adolescent human tendon tissue contains a population of cells with intrinsic differentiation potential.

**Methods:**

Cells derived from adolescent non-degenerative hamstring tendons were characterized by immunohistochemistry and FACS-analysis. Cells were cultured for 21 days in osteogenic, adipogenic, and chondrogenic medium and phenotypical evaluation was carried out by immunohistochemical and qPCR analysis. The results were compared with the results of similar experiments on adult bone marrow-derived stromal cells (BMSCs).

**Results:**

Tendon-derived cells stained D7-FIB (fibroblast-marker) positive, but α-SMA (marker for smooth muscle cells and pericytes) negative. Tendon-derived cells were 99% negative for CD34 (endothelial cell marker), and 73% positive for CD105 (mesenchymal progenitor-cell marker). In adipogenic medium, intracellular lipid vacuoles were visible and tendon-derived fibroblasts showed upregulation of adipogenic markers FABP4 (fatty-acid binding protein 4) and PPARG (peroxisome proliferative activated receptor γ). In chondrogenic medium, some cells stained positive for collagen 2 and tendon-derived fibroblasts showed upregulation of collagen 2 and collagen 10. In osteogenic medium Von Kossa staining showed calcium deposition although osteogenic markers remained unaltered. Tendon-derived cells and BMCSs behaved largely comparable, although some distinct differences were present between the two cell populations.

**Conclusion:**

This study suggests that our population of explanted human tendon cells has an intrinsic differentiation potential. These results support the hypothesis that there might be a role for altered tendon-cell differentiation in the pathophysiology of tendinosis.

## Background

Tendinosis is a chronic degenerative tendon disorder occurring particularly among athletes and middle-aged people [1]. As its pathophysiology is still largely unknown, only symptomatic treatment options are available, with limited success rates [1,2]. A better understanding of the cellular processes involved in the development of tendinosis lesions may ultimately improve treatment and prevention.

Histopathological findings in tendinosis have been described in detail [3,4]. In brief, hypercellularity and rounding of the cell nuclei indicate a relatively high metabolic activity. Likewise, altered extracellular matrix composition reflects changes in cellular behaviour. For instance, in tendinosis lesions there is a higher amount of glycosaminoglycans [3]. Lipid accumulation and calcium deposition have also been described [5]. Thus, the histopathological findings may indicate the presence of cells with diverse phenotypes, different from that of tenocytes under healthy conditions.

Cells with multilineage differentiation potential likely play an important role in the body's capacity to naturally remodel, repair, and regenerate various tissue types where necessary [6]. However, the multilineage differentiation potential of cells might also be involved in pathological processes. Although the pathophysiology of tendinosis is largely unclear, histological findings suggest that multipotent cells might be implicated in its development. The origin of these multipotent cells is unknown. They may be recruited from the bone marrow in response to tendon tissue injury, and migrate through the circulation to the site of tissue damage [7]. They might also be present in the tendon tissue itself.

Local progenitor cells with multilineage potential have previously been found in many locations within the musculoskeletal system, e.g. in bone marrow, skin, periosteum, bone, muscle and adipose tissue [8-15]. On the other hand, progenitor cells are not the only cells with multilineage potential: some highly differentiated cells are capable of transdifferentiation, i.e. switching their phenotype to another lineage. This transdifferentiation has been demonstrated for highly differentiated chondrocytes [16,17].

Multipotent cells have been found in virtually all tissues of the musculoskeletal system, but it is not known if tendon tissue has a cell population with multilineage potential. In this study, we investigated whether the population of cells derived from non-degenerative tendon tissue has differentiation potential similar to bone marrow-derived stromal cells (BMSCs). Specifically, we characterized human tendon-derived fibroblasts by immunohistochemical staining and FACS-analysis. Then, after a culture period of 21 days in adipogenic, chondrogenic, and osteogenic medium, we evaluated changes in their phenotype using immunohistochemical and histochemical stainings as well as gene expression analysis.

## Methods

### Study design

Cells were explanted from human adolescent non-degenerative hamstring tendon tissue (n = 5). After the phenotype of the cells was analyzed by immunohistochemical staining and FACS-analysis, cells were cultured for 21 days on osteogenic, adipogenic, or chondrogenic medium. The differentiation potential of the tendon-derived cell population was evaluated by immunohistochemical and histochemical staining and real-time RT-PCR, and was compared with the differentiation potential of human femoral-shaft-derived BMSCs (n = 5).

### Isolation of tendon-derived cells and BMSCs

Human tendon-derived cells were cultured from explants from hamstring tendon tissue of five adolescents (age 12–17 years) undergoing hamstring-tendon release for treatment of knee-contractures (MEC-2006-069). In this clinical condition the tendon is primarily not affected, but is exposed to continuously high tensile strains. After the peritendineum had been carefully removed, the tendon was cut into 3 mm^3 ^sections, transferred into six-well plates (Corning, NY, USA) and cultured in expansion medium (Dulbecco's modified Eagle's medium, 10% fetal calf serum (FCS), 50 μg/ml gentamicin and 1.5 μg/ml fungizone (all Invitrogen, Scotland, UK)). Tissue cultures were maintained at 37°C in a humidified atmosphere of 5% CO_2 _for ten days, with three medium changes. During this time, fibroblasts migrated out of the tissue and adhered to the bottom of the culture dish. Cells were subcultured and trypsinized at subconfluency and cells from the third to the fifth passage were used for the differentiation experiments.

Human bone marrow stromal cells (BMSCs) were isolated from femoral shaft biopsies of six patients (age 42–72 years) undergoing total hip replacement for treatment of osteoarthritis (MEC-2004-142). BMSCs were isolated from aspirated marrow acccording to procedures described earlier [18]. Briefly, heparinized femoral-shaft marrow aspirate was plated out and after 24 hours, non-adherent cells were removed with 2% FCS in 1×PBS. Adherent cells were subcultured in medium with 10% FCS and trypsinized at subconfluency. Cells from the second to the fourth passage were used for the differentiation experiments. The used serum lot was selected specifically for the maintenance of multipotential cells.

### Phenotypic characterization of tendon-derived cells

Explants harvested on day 6 of the explantation period were fresh frozen in liquid nitrogen and 6 μm frozen sections were fixated in acetone. Cells in monolayer cultures, passage 1 and 4, were fixated in ice-cold 70% ethanol.

#### Ki-67, D7-FIB, and α-SMA staining

Cells and histological sections were incubated with either mouse monoclonal antibody against 11-fibrau (Clone D7-FIB; diluted 1:400; Imgen, Netherlands), a marker for fibroblasts [19], or monoclonal antibody against α-SMA (Clone 1A4; diluted 1:1000; Sigma, St.Louis, Missouri, USA), a marker for smooth muscle cells and pericytes [20], for two hours. Cells were rinsed in 1×PBS and IHC detection was performed using Link-Label (Biotin-based) Multilink^® ^IHC Detection Kit (Biogenex, San Ramon, CA). Finally, a new fuchsin substrate was added to obtain a pink signal in positive cultures. Cells were counterstained with Gill's haematoxilin (Sigma). For Ki-67 staining histological sections were pre-incubated in 1% H_2_O_2 _(Sigma) in methanol (Sigma) and then incubated with mouse monoclonal antibody Ki-67 (M7187; diluted 1:25; Dako, Glostrup, Denmark). IHC detection was performed using StrAviGen Multilink^® ^Kit (Biogenex, San Ramon, CA), substrate development was performed using the SK-4800 Vector^® ^NovaRED™ Substrate kit (Vector Laboratories, Burlingame, CA), and no counterstaining was performed.

#### FACS-analysis

Trypsinized first to fifth passage cells were incubated at 4°C for 30 minutes with saturating amounts of human antibodies CD105-PE (dilution 1:20; BD Biosciences, San Jose, USA), a marker for mesenchymal progenitor cells [21], and CD34-PE (dilution 1:20; Ancell, Bayport, USA), a marker that remains negative in non-hematogenic progenitor cells [20] and is positive for hematogenic progenitor cells, endothelial cells, and pericytes [22-24]. Cells were washed and resuspended in 300 μl HBN buffer (Hank's Balanced Salt Solution (HBSS; GIBCO, Breda, The Netherlands) + 0.5% (wt/vol) Bovine Serum Albumin + 0.05% (wt/vol) sodium azide) and analyzed by flow cytometric analysis using a FACSCalibur flow cytometer and Cellquest software (BD Biosciences, San Jose, USA) with a minimum of 10,000 events acquired.

### Differentiation experiment

After trypsinisation, cells were seeded in six-well plates and cultured in a modified version of three differentiation media described earlier [18]. Briefly, cells were seeded at 3,000 cells/cm^2 ^to induce osteogenic differentiation and then cultured in an osteogenic induction medium containing DMEM plus 10% FCS and freshly added β-glycerophosphate 10 mM (Sigma, St. Louis, USA), dexamethasone 0.1 μM (Sigma) and L-ascorbic acid 2 phosphate 0.5 mM (Sigma). Cells were seeded at 20,000 cells/cm^2 ^to induce adipogenic differentiation, and cultured in adipogenic induction medium containing DMEM with 10% FCS, supplemented with dexamethasone 1 μM, indo-methacin 0.2 mM, insulin 0.01 mg/ml, and 3-isobutyl-l-methyl-xanthine 0.5 mM (all from Sigma). To induce chondrogenic differentiation, cells were cultured in 1.2% low viscosity alginate beads at a density of 4 × 10^6 ^cells/ml in serum-free chondrogenic induction medium containing DMEM supplemented with TGF-β2 10 ng/ml (R&D Systems, UK), L-ascorbic acid 2 phosphate 25 μg/ml (Sigma), sodium pyruvate 100 μg/ml (Invitrogen), proline 40 μg/ml (Sigma) and ITS+ (diluted 1:100; BD Biosciences, Bedford, MA). All media contained 50 μg/ml gentamicin and 1.5 μg/ml fungizone. Cells were cultured in differentiation media for 21 days, with media changes twice a week. On day 21 of culture, two wells were harvested for RNA extraction and one well was used for histochemical evaluation. One well was cultured for 21 days on expansion medium as control condition for the histochemical stainings.

### Gene expression analysis

At harvesting, monolayer cell cultures were suspended in RNA-Bee™ (TEL-TEST, Friendswood, TX, USA). Alginate beads were dissolved in 150 μl of 55 mM sodium citrate in 150 mM sodium chloride per bead (both Fluka, Steinheim, Switzerland) and cell pellets were subsequently suspended in RNA-Bee™. RNA was precipitated with 2-propanol, purified with lithium chloride, and 1 μg total RNA of each sample was reverse-transcribed into cDNA using RevertAid™ First Strand cDNA Synthesis Kit (MBI Fermentas, St. Leon-Rot, Germany). Primers were designed using PrimerExpress 2.0 software (Applied Biosystems, Foster City, CA, USA) to meet Taqman^® ^or SYBR^®^Green requirements and were designed to bind to separate exons to avoid co-amplification of genomic DNA. BLASTN ensured gene specificity of all primers listed in Table 1. As osteogenic markers osterix (SP7), RUNT-related transcription factor 2 (RUNX2), and osteocalcin (BGLAP) were studied, while SOX9, aggrecan (AGC1), collagen 2 (COL2A1), and collagen 10 (COL10A1) were used as chondrogenic markers. Adipogenic markers studied were fatty acid binding protein 4 (FABP4) and peroxisome proliferative activated receptor γ (PPARG). Amplifications were performed as 25 μl reactions using either TaqMan^® ^Universal PCR MasterMix (ABI, Branchburg, New Jersey, USA) or qPCR™ Mastermix Plus for SYBR^®^Green I (Eurogentec, Nederland B.V., Maastricht, The Netherlands) according to the manufacturer's guidelines. Real-Time RT-PCR (QPCR) was done using an ABI PRISM^® ^7000 with SDS software version 1.7. Data were normalized to GAPDH which was stably expressed across sample conditions (not shown). Relative expression was calculated according to the 2^-ΔCT ^formula [25] using averages of duplo samples.

Statistical analysis on averages of duplo samples was performed using SPSS 11.5 software (SPSS Inc., Chicago, IL, USA). Groups on differentiation media were compared with a Kruskall-Wallis H test and post-hoc Mann-Whitney U test. For both tests p < 0.05 was considered to indicate statistically significant differences. The graphs are Box-Whisker plots, with the box representing the middle two quartiles (25–75) and the Whiskers the highest and lowest value. All outlier variables were included in the statistical analyses but excluded in the graphical display.

### Histochemical and immunohistochemical stainings

#### Von Kossa staining

Cells were fixed in formalin, hydrated in milliQ water, immersed in 5% silver nitrate solution (Sigma) for 10 minutes, rinsed and exposed to light for 10 minutes. Excess silver nitrate was removed with 5% sodium-thiosulphate (Sigma) and cells were rinsed in distilled water, followed by a counterstaining with azophloxine (Sigma).

#### Oil Red O staining

Cells were fixed in 10% formalin, treated with 0.3% Oil red O solution (Sigma) for 15 min, and then repeatedly washed with tap water.

#### Collagen type 2 staining

Alginate beads were dissolved in sodium citrate, cytospins were prepared and stored at -80°C. Cytospins were fixed in acetone and treated with 1% hyaluronidase (Sigma) for 20 min. Cell monolayers were fixed with 70% ethanol, treated with 50 mM NH_4_Cl (Sigma), and permeabilised in a 0.1% Triton X-100 (Sigma) solution. Cells were incubated with mouse monoclonal antibody against collagen type 2 (II-II6B3, diluted 1:100; Developmental Studies Hybridoma Bank) for 2 hours. Anti-mouse Fab fragments conjugated with alkaline phosphatase (GAMAP, diluted 1:100; Immunotech, Marseille, France) were added. Finally, alkaline phosphatase conjugated anti-mouse antibodies in combination with a new fuchsin substrate were added to obtain a pink signal in positive cultures. Counterstaining with Gill's haematoxilin (Sigma) was performed.

## Results

### Characterization of tendon-derived fibroblasts

Histological examination of the adolescent hamstring tendon explants confirmed normal tissue morphology. Specifically, no degenerative lesions, inflammatory cell infiltration, (partial) ruptures, chondroid metaplasia, or calcifications were seen.

**Figure 1:**
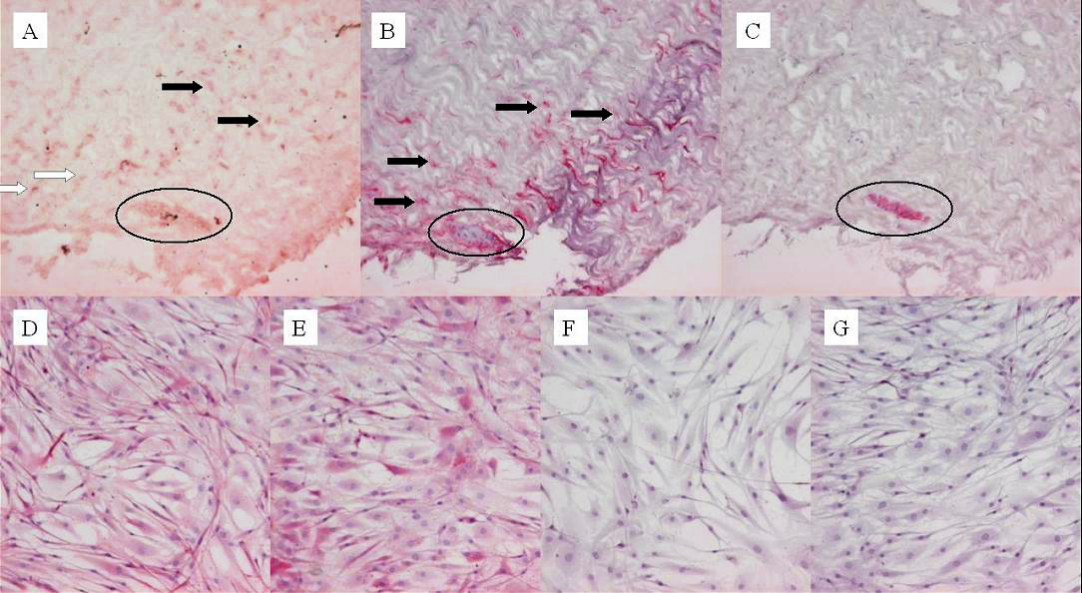
Ki-67, D7-FIB, and α-SMA staining on tendon explants (day 6 of explantation period) and on tendon-derived fibroblasts (TDF) in monolayer culture. Ki-67 positive (proliferating) cells in the explants were located in the tendinous tissue (A, black arrow), in the endotenon (A, white arrow), and in the vascular walls (A, circle). Cells in the tendon tissue and in the endotenon stained positive for fibroblastmarker D7-FIB (B). Cells in the vascular walls remained negative for D7-FIB (B) and instead stained positive for α-SMA, a marker for pericytes and smooth muscle cells (C). All TDFs in monolayer culture stained positive for D7-FIB from passage one (D) to passage four (E) and remained negative for α-SMA from passage one (F) to passage four (G).

During the explant culture period, proliferating cells (Ki-67 positive) were located between the highly organized collagen fibres of the tendon tissue and also in the connective tissue of the endotenon. These cells stained positive for fibroblast-marker D7-FIB. On the other hand, proliferating cells were also seen in the vascular walls, staining negative for D7-FIB but positive for α-SMA, a marker for pericytes and smooth muscle cells (Figure 1).

Cells explanted from the tendon tissue had a characteristic spindle-shaped fibroblastic morphology. Through the first four passages in monolayer culture all tendon-derived cells stained positive for D7-FIB but stained negative for α-SMA (Figure 1). On further characterization by FACS-analysis 99.1 +/- 1.1 % of the tendon-derived fibroblasts were CD34 negative and 72.6 +/- 22.9 % were CD105 positive (average of passage 2 to 5 tendon-derived cells, n = 4). The BMSCs had 99.7 +/- 0.4 % CD34 negative cells and 93.8 +/- 4.6 % CD105 positive cells (average of passage 1 to 5 BMSCs, n = 8).

### Adipogenic markers

**Figure 2:**
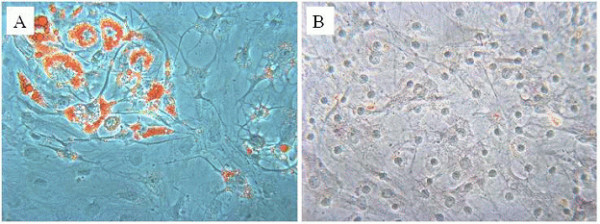
Oil Red O staining on tendon-derived fibroblasts cultured for 21 days in adipogenic medium (A) (note that not all cells but merely clusters of cells formed Oil Red O positive lipid vacuoles inside the cell's main body) or in control medium (B). Like cells in control medium, cells cultured in osteogenic or chondrogenic medium were negative (figures not shown).

Light microscopy revealed the presence of vacuoles within approximately one third of the cells in all adipogenic cultures of tendon-derived fibroblasts. Oil Red O staining confirmed that these were lipid vacuoles (Figure 2A). Only cells aggregated into clusters stained positively for lipid vacuoles. Tendon-derived fibroblasts cultured on control medium (Figure 2B), on osteogenic, or on chondrogenic medium (not shown) did not develop any lipid vacuoles. Cellular distribution of Oil Red O positive BMSCs cultured in adipogenic medium was more homogenous with approximately 75% of cells staining positively (results not shown).

**Figure 3:**
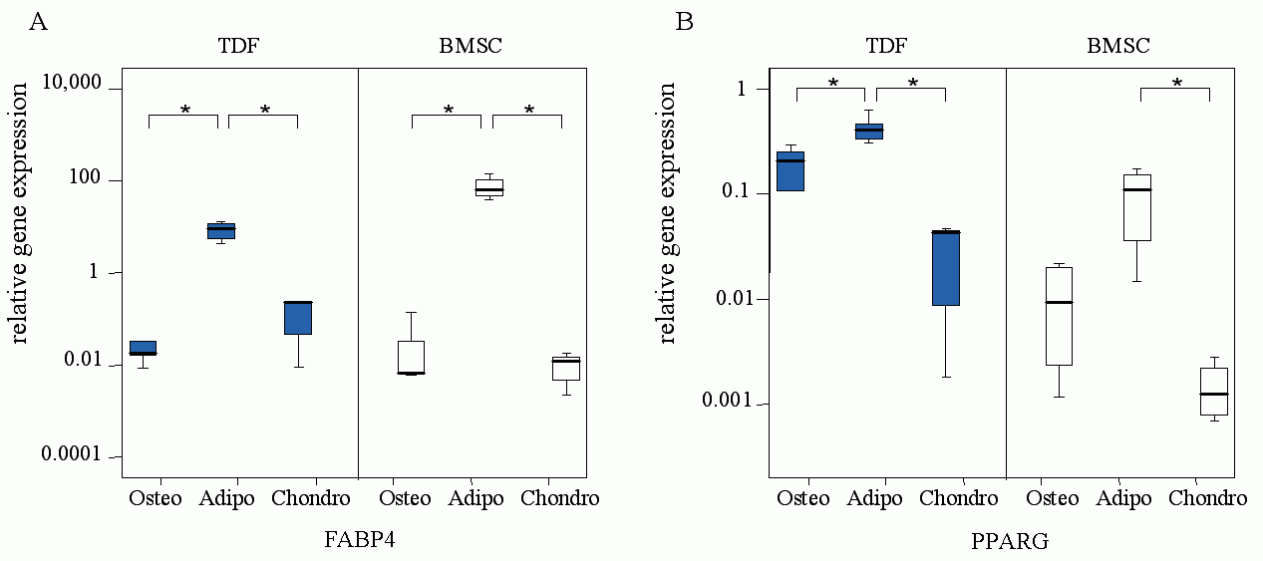
Expression levels of adipogenic markers in tendon-derived fibroblasts (TDF) and bone marrow-derived stromal cells (BMSC). Cells were cultured for 21 days on osteogenic (*N *= 5 for TDF, *N *= 4 for BMSC), adipogenic (*N *= 5 for TDF, *N *= 5 for BMSC), or chondrogenic (*N *= 3 for TDF, N = 5 for BMSC) induction medium. The relative, GAPDH-normalized, expression levels of fatty acid binding protein 4 (FABP4)(A) and peroxisome proliferator activated receptor γ (PPARG)(B) is displayed on the vertical axis. * Indicates a *P*-value<0.05.

In addition to this, culture of tendon-derived fibroblasts in adipogenic medium significantly upregulated expression of FABP4 and PPARG compared to those cultured in osteogenic (both p = 0.009) and chondrogenic medium (both p = 0.025). Similar findings were seen in the BMSC cultures although the difference in PPARG expression between the osteogenic and adipogenic medium condition did not reach statistical significance in the BMSC cultures (Figure 3). In the BMSC cultures PPARG expression was significantly higher in adipogenic medium compared to chondrogenic medium (p = 0.021); FABP4 expression was upregulated in the adipogenic medium compared to osteogenic medium (p = 0.021) and chondrogenic medium (p = 0.021).

### Chondrogenic markers

**Figure 4:**
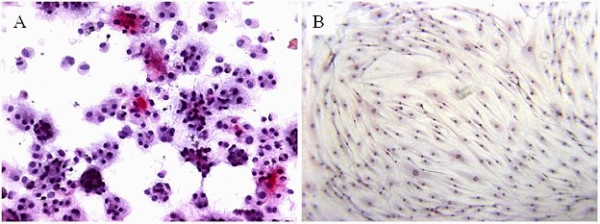
Immunohistochemical staining for collagen type 2 on tendon-derived fibroblasts. 5% of the cells cultured for 21 days in alginate beads in chondrogenic medium stained positive (A). Cells cultured in monolayer in control medium remained negative (B) as did cells in adipogenic or osteogenic media (figures not shown).

Immunohistochemical staining for collagen type 2 was performed on tendon-derived fibroblasts cultured in chondrogenic, adipogenic, osteogenic, and control medium for 21 days. In all chondrogenic medium conditions approximately 5% of the cells stained positive for collagen type 2 (Figure 4A). Tendon-derived fibroblasts cultured in control medium (Figure 4B), as well as adipogenic and osteogenic medium were immunonegative for collagen type 2 (not shown). BMSC cultures showed a similar amount of collagen type 2 staining in chondrogenic medium (not shown).

**Figure 5:**
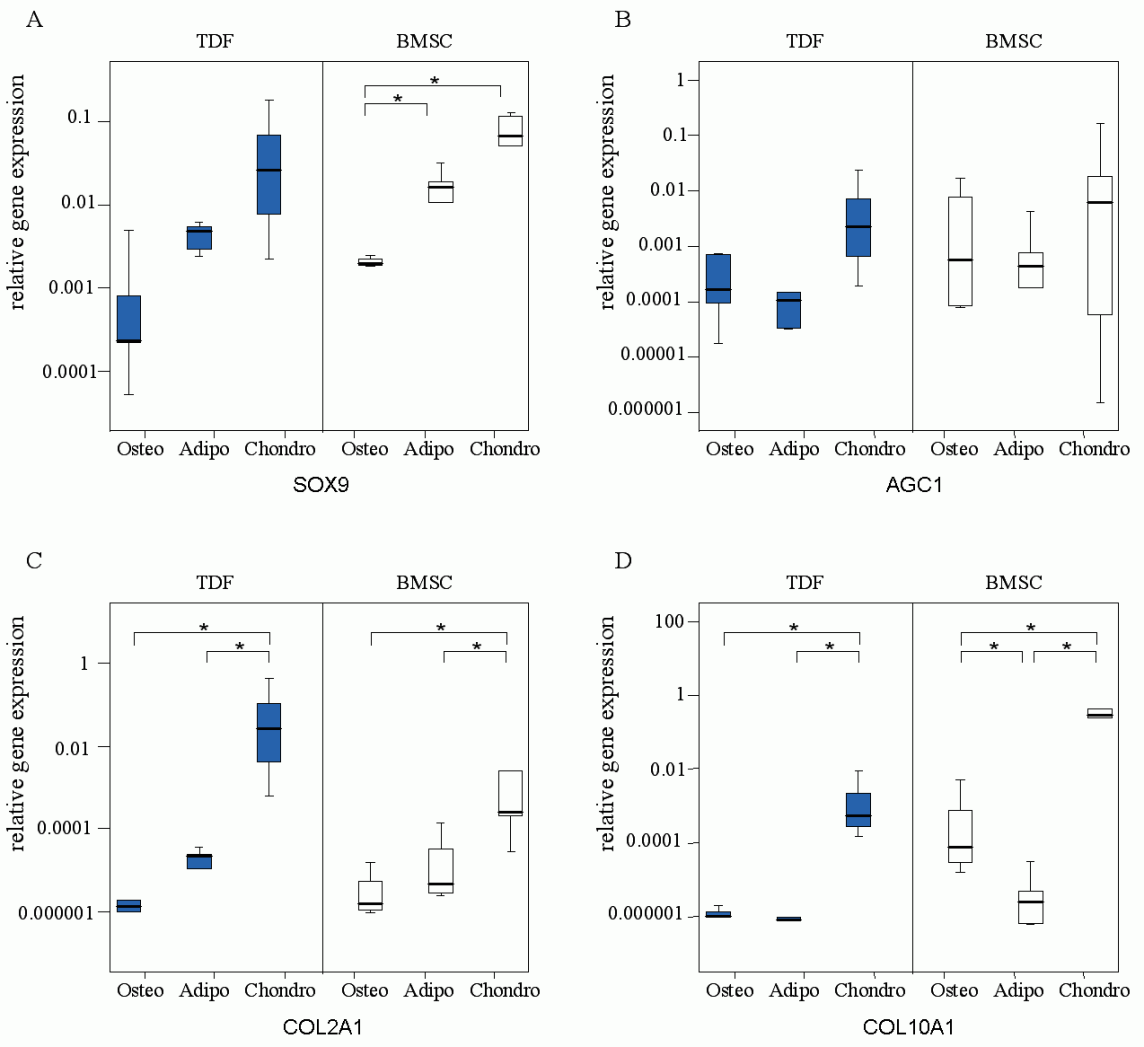
Expression levels of chondrogenic markers in tendon-derived fibroblasts (TDF) and bone marrow-derived stromal cells (BMSC). SOX9 (A), aggrecan (AGC1)(B), collagen 2 (COL2A1)(C) and collagen 10 (COL10A1)(D). See figure 3 for reminder of key.

Culture of tendon-derived fibroblast in chondrogenic medium significantly increased expression of chondrogenic markers COL2A1 and COL10A1 (the latter is considered to be a marker for hypertrophic cartilage formation) compared to the osteogenic condition (p = 0.025 for both genes) and adipogenic condition (p = 0.025 for both genes). Expression of SOX9 and AGC1 in chondrogenic medium compared to osteogenic and adipogenic medium was not significantly different (Figure 5).

BMSC cultures also showed a significantly higher expression of COL2A1 and COL10A1 in chondrogenic medium compared to osteogenic medium (p = 0.014 for both genes) and adipogenic medium (p = 0.028 for COL2A1 and p = 0.009 for COL10A1). SOX9 expression in BMSCs showed the same trend as in the tendon-derived fibroblast cultures, but the differences only reached significance in the BMSC cultures (osteogenic versus adipogenic medium p = 0.014 and osteogenic versus chondrogenic medium p = 0.014). Expression of AGC1 in the BMSCs did not differ significantly between the three medium conditions. Interestingly, BMSCs cultured in osteogenic medium had significantly upregulated COL10A1 compared to the adipogenic condition (p = 0.027). This phenomenon was not seen in the tendon-derived fibroblasts (Figure 5).

### Osteogenic markers

**Figure 6:**
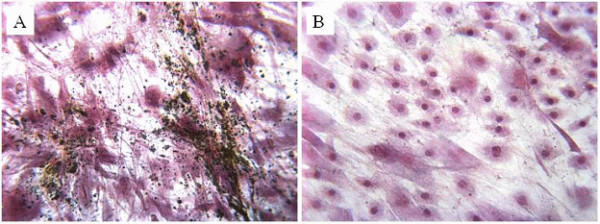
Von Kossa staining on tendon-derived fibroblasts cultured for 21 days in osteogenic (A) or control medium (B). Calcium deposition was seen in osteogenic medium (A), not in control medium (B) or in adipogenic or chondrogenic media (figures not shown).

Von Kossa staining of tendon-derived fibroblasts in the osteogenic condition showed clustered areas of calcium deposition, whereas the tendon-derived fibroblast cultures in control medium had no calcium deposition (Figure 6). Also, tendon-derived fibroblast cultures in adipogenic and chondrogenic medium remained negative for calcium (results not shown). Similarly, in BMSC cultures, calcium deposition was found only in the osteogenic condition (not shown).

**Figure 7:**
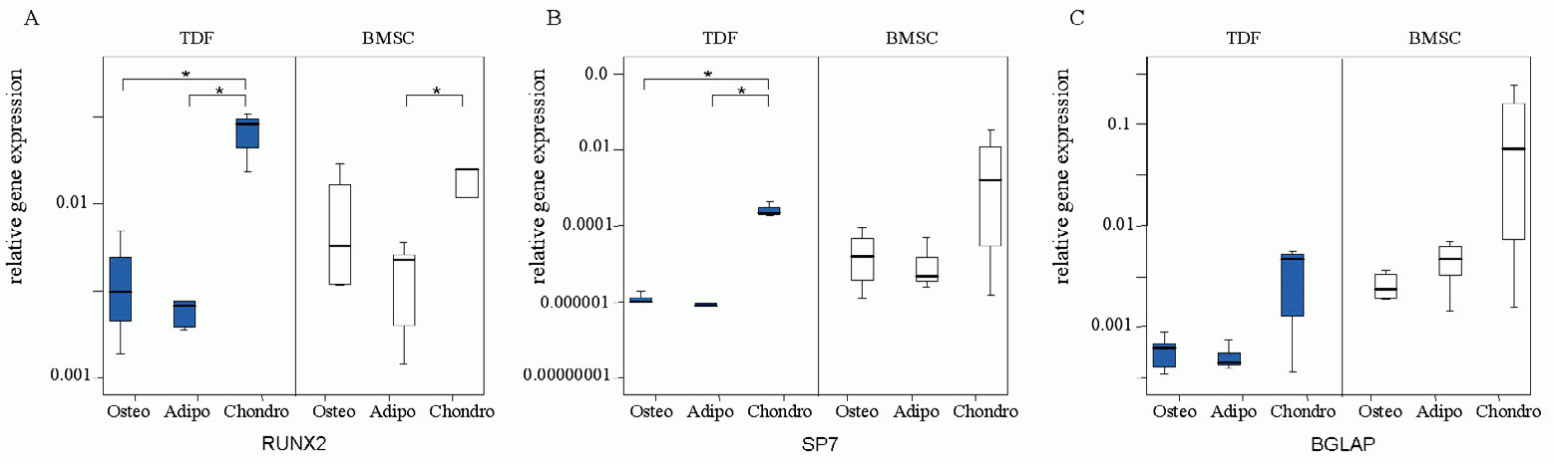
Expression levels of osteogenic markers in tendon-derived fibroblasts (TDF) and bone marrow-derived stromal cells (BMSC). RUNT-related transcription factor 2 (RUNX2) (A), SP7 (B), and BGLAP (C). See figure 3 for reminder of key.

Although we did find expression of osteogenic markers RUNX2, osterix, and osteocalcin, culture of tendon-derived fibroblasts in osteogenic medium did not induce statistically significant upregulation of any of these genes. Similar results were found by QPCR of these markers in BMSCs cultured in osteogenic medium (Figure 7). In the tendon-derived fibroblast cultures SP7 and RUNX2 (both also known to play an important role in chondrogenic differentiation and hypertrophic cartilage formation [26]) were significantly upregulated in the chondrogenic medium compared to the osteogenic (p = 0.025 for both genes) and adipogenic medium (p = 0.025 for both genes)(Figure 7). BMSCs also showed an upregulation of SP7 and RUNX2 in the chondrogenic medium. RUNX2 upregulation was significant (p = 0.016 for the difference in gene expression of RUNX2 between adipogenic and chondrogenic medium in BMCSs), but SP7 upregulation in chondrogenic medium did not reach significance (Figure 7). In summary, chondrogenic medium not only stimulated expression of chondrogenic marker COL2A1, but also of COL10A1, RUNX2, and SP7.

## Discussion

This in-vitro differentiation study suggests that a proportion of the cell population explanted from adolescent human tendon tissue may have adipogenic and chondrogenic differentiation potential. In adipogenic medium lipid vacuoles were visible and tendon-derived fibroblasts showed upregulation of FABP4 and PPARG. In chondrogenic medium, positive collagen 2 staining was visible around some of the tendon-derived fibroblasts and the tendon-derived fibroblasts showed upregulation of COL2A1 and COL10A1. In osteogenic medium Von Kossa staining showed calcium deposition, although osteogenic markers remained unaltered, as assessed by qPCR. Compared to the BMSCs, the diffentiation capacity of our tendon-derived fibroblasts was similar, although some differences were visible, mainly concerning the number of Oil Red O positive cells.

To our knowledge, this is the first study evaluating the intrinsic differentiation potential of human tendon cells in vitro. Previously, Salingcarnboriboon et al [27] established three murine tendon cell lines by clonal expansion and showed that these single cell clones could differentiate towards multiple mesenchymal lineages upon culture in appropriate differentiation media. Therefore, they suggested that cells with mesenchymal stem-cell-like characteristics might exist in murine tendon tissue. Our experiments cannot distinguish between individual cells with multilineage potential and a cell population containing more or less strongly committed cells. We did find that not all of the tendon-derived fibroblasts appeared to be capable of differentiating towards other lineages, e.g. not all fibroblasts but merely clusters of fibroblasts created lipid vacuoles in adipogenic medium and only a small proportion of approximately 5% of the cells stained positive for collagen type 2. In addition to this observation, only a subpopulation of 72.6 +/- 22.9 % of these tendon-derived fibroblasts stained positive for CD105 and this subpopulation might be responsible for the observed differentiation potential.

Due to their spindle-shaped morphology in monolayer culture and because all explanted cells stained D7-FIB positive in passage one through passage four, we identified these cells as tendon-derived fibroblasts. Based on the results of the Ki-67 staining, it could be surmised that this mixed population may be partly derived from the tendon tissue and partly from the endotenon. It is possible that these cells were already preselected for during the explantation procedure, based on cellular motility, chemotactic responses or plastic adherence characteristics. Within this culture population, mature tendon-derived fibroblasts with transdifferentiation capacity or a specific subpopulation of tendon-derived progenitor cells might exist. Several authors have found that pericytes isolated from different tissues can be induced to differentiate into various connective tissue phenotypes [8]. It seems unlikely that the presence of vascular pericytes in tendon tissue, which might be another multipotent cell source in tendon tissue [28], can account for our findings. Not only is tendon a poorly vascularized tissue, but also the tendon-derived fibroblasts remained negative for pericyte marker α-SMA through the first four passages. Furthermore, our explanted cell population was 98.5 +/- 0.7 % negative for CD34 on FACS-analysis. It seems unlikely that the small portion of 1.5% CD34-positive tendon-derived fibroblasts accounts for the results of the immunohistochemical staining and the changes in gene expression pattern.

A cell population with multilineage potential that might be present in tendon tissue, is likely involved in tendon repair. Such a population might also contribute to the development of tendinosis, as this tendon disorder is associated with fatty degeneration, glycosaminoglycan accumulation, and calcifications. In addition to these internal multipotent cells other cells with multilineage potential may arrive at the site of overuse or tendon damage through the vascular system and contribute to the development or repair of tendinosis: upregulation of VEGF was found in human achilles tendinosis lesions [29] and VEGF can act as a chemotactic stimulus for mesenchymal cells [30]. In-vivo control of differentiation of cells with multilineage potential might prove useful in the future for prevention of tendinosis lesions or induction of in-situ repair of these lesions.

The exact changes in the tendon microenvironment outside the cells that play a role in cellular differentiation are still the subject of many investigations. First, the capability of specific growth factors, cytokines, and other inflammatory mediators to influence the cellular differentiation process has been demonstrated. Changes in the concentration of various growth factors have also been found in tendinosis lesions: for instance, a higher number of cells expressing TGF-β2 and TGF-βRII (a TGF-β receptor) in chronic achilles tendinosis lesions [31] and increased expression of TGF-β1 in patellar tendinosis [32] have been reported. TGF-β molecules are also used in vitro to induce chondrogenic differentiation of mesenchymal progenitor cells [21]. Second, changes in the degree of vascularization of the tissue, as reported in achilles tendinosis lesions [33], might influence the tendon cell differentiation state in vivo. For instance, oxygen tension influences the redifferentiation potential of dedifferentiated chondrocytes in vitro [34] and hypoxia not only promotes the differentiation of bone mesenchymal stem cells along a chondrocyte pathway [35], but can also promote the formation of an adipocyte-like phenotype with cytoplasmic lipid inclusions in human MSCs [36]. Third, following repetitive tendon overload and its resulting microruptures in tendinosis lesions [37], tendon cells may experience an altered mechanical microenvironment, which in turn might influence chondrogenic, osteogenic, or tenogenic differentiation [38].

Our findings demonstrate that an intrinsic differentiation capacity is present in tendon tissue of adolescent individuals. However, age plays an important role in the response of musculoskeletal tissues in response to environmental changes. It has been demonstrated that adult but not juvenile cartilage has lost its ability to regenerate (cited by Hunter [39]) and BMSCs gradually loose their differentiation potential as subjects grow older [40]. Therefore, the adolescent tendon samples used in this study might not be representative of tendon tissue in adult tendinosis lesions. Since tendon cell populations derived from adult and from late fetal equine tendons have demonstrated similar levels of a weak progenitor cell ability [41], it might be justified to speculate that tendon-derived fibroblasts from older subjects may still have some differentiation capacity. However, this certainly needs further investigation.

A tendon-cell population with intrinsic differentiation capacity might be used in vivo for repair of lesions and might play a role in tendinosis. However, extrapolating results from in-vitro cultures to the in-vivo situation must be done with tremendous caution, particularly as the expansion-culture period prior to experimentation may have led to the loss of the original tendon fibroblast phenotype (due to dedifferentiation): the latter being well known in chondrocyte-cultures [42]. Whether cells in vivo can be stimulated to display this differentiation potential remains to be elucidated.

## Conclusion

Obtaining insight in the cellular behaviour and pathogenesis in tendinosis is crucial in order to develop mechanism-based therapies. Our study suggests that adolescent tendon tissue has an intrinsic differentiation potential. This study conducted on human tenocytes corroborates the findings that cells with mesenchymal stem-cell-like characteristics might exist in murine tendon tissue. Our results support the hypothesis that altered tendon-cell differentiation might play a role in the pathophysiology of tendinosis.

## Abbreviations

AGC1 = aggrecan

BGLAP = osteocalcin

BMSC = bone marrow-derived stromal cell

COL10A1 = collagen type 10, alpha 1 chain

COL2A1 = collagen type 2, alpha 1 chain

D7-FIB = 11-fibrau, fibroblast antibody

DMEM = Dulbecco's modified Eagle's medium

FABP4 = adipocyte fatty acid binding protein 4

FACS = fluorescense activated cell sorter

FCS = fetal calf serum

HBN buffer = Hank's Balanced Salt Solution (HBSS; GIBCO, Breda, The Netherlands) + 0.5% (wt/vol) Bovine Serum Albumin + 0.05% (wt/vol) sodium azide

IHC = immunohistochemistry

Ki-67 = a protein strictly associated with cell proliferation

MEC = medical ethical committee

SOX9 = SRY-box 9

PPARG = peroxisome proliferative activated receptor gamma

QPCR = quantitative polymerase chain reaction

RT-PCR = reverse transcriptase polymerase chain reaction

RUNX2 = RUNT-related transcription factor 2

SP7 = Sp7 transcription factor/osterix

TDF = tendon-derived fibroblast

α-SMA = alpha smooth muscle actin

## Competing interests

The author(s) declare that they have no competing interests.

## Authors' contributions

MM performed the study, performed the statistical analysis, and drafted the manuscript. JLK participated in the design of the study and in the culture work. HJ designed primers and probes, helped with the molecular biology, and helped to draft the manuscript. MMAV: performed the FACS analysis. MPH was involved in the study design and conception and helped to draft the manuscript. NK participated in the culture work and immunohistological staining. JPTML participated in the design of the study and helped to draft the manuscript. HW was involved in the study design and conception, participated in the design of the study, and helped to draft the manuscript. JANV was involved in the study design and conception and helped to draft the manuscript. GJVMO was involved in the study design and conception, and helped to draft the manuscript. All authors read and approved the manuscript.

## Pre-publication history

The pre-publication history for this paper can be accessed here:



## Figures and Tables

**Table 1:** Primer and probe nucleotide sequences of the tested genes

Gene	Accession no.	Primer	Probe
GAPDH	NM_002046.2	F: ATGGGGAAGGTGAAGGTCG R: TAAAAGCAGCCCTGGTGACC	CGCCCAATACGACCAAATCCGTTGAC
RUNX2	NM_001024630.1 NM_00101505.1 NM_0043468.3	F: GCCTTCAAGGTGGTAGCCC R: CGTTACCCGCCATGACAGTA	CCACAGTCCCATCTGGTACCTCTCCG
BGLAP	NM_199173.2	F: GAAGCCCAGCGGTGCA R: CACTACCTCGCTGCCCTCC	TGGACACAAAGGCTGCACCTTTGCT
PPARG*	NM_138712.2 NM_015869.3 NM_005037.4 NM_138711.2	F: AGGGCGATCTTGACAGGAAA R: TCTCCCATCATTAAGGAATTCATG	
SOX9	NM_000346.2	F: CAACGCCGAGCTCAGCA R: TCCACGAAGGGCCGC	TGGGCAAGCTCTGGAGACTTCTGAACG
AGC1	NM_001135.1 NM_013227.1	F:TCGAGGACAGCGAGGCC R:TCGAGGGTGTAGCGTGTAGAGA	ATGGAACACGATGCCTTTCACCACGA
COL2A1	NM_033150	F: GGCAATAGCAGGTTCACGTACA R: CGATAACAGTCTTGCCCCACTT	CCGGTATGTTTCGTGCAGCCATCCT
COL10A1	NM_000493.2	F: CAAGGCACCATCTCCAGGAA R: AAAGGGTATTTGTGGCAGCATATT	TCCAGCACGCAGAATCCATCTGA

F: forward; R: reverse; * SYBR^®^Green assay. Commercially available, so-called assays-on-demand (Applied Biosystems, Foster City, CA, U.S.A.) were used to detect osterix (SP7; Hs_00541729_m1) and fatty acid binding protein 4 (FABP4; Hs_00609791)-specific mRNA.
